# Plant endemism in the Sierras of Córdoba and San Luis (Argentina): understanding links between phylogeny and regional biogeographical patterns^1^

**DOI:** 10.3897/phytokeys.47.8347

**Published:** 2015-03-17

**Authors:** Jorge O. Chiapella, Pablo H. Demaio

**Affiliations:** 1 Instituto Multidisciplinario de Biología Vegetal (IMBIV-Conicet-UNC). Vélez Sarsfield 299 - X5000JJC Córdoba – Argentina

**Keywords:** Argentina, Sierras of Córdoba and San Luis, endemics, phylogenies, Argentina, Sierras de Córdoba y San Luis, endemismos, filogenias

## Abstract

We compiled a checklist with all known endemic plants occurring in the Sierras of Córdoba and San Luis, an isolated mountainous range located in central Argentina. In order to obtain a better understanding of the evolutionary history, relationships and age of the regional flora, we gathered basic information on the biogeographical and floristic affinities of the endemics, and documented the inclusion of each taxon in molecular phylogenies. We listed 89 taxa (including 69 species and 20 infraspecific taxa) belonging to 53 genera and 29 families. The endemics are not distributed evenly, being more abundant in the lower than in the middle and upper vegetation belts. Thirty-two genera (60.3%) have been included in phylogenetic analyses, but only ten (18.8%) included local endemic taxa. A total of 28 endemic taxa of the Sierras CSL have a clear relationship with a widespread species of the same genus, or with one found close to the area. Available phylogenies for some taxa show divergence times between 7.0 – 1.8 Ma; all endemic taxa are most probably neoendemics *sensu* Stebbins and Major. Our analysis was specifically aimed at a particular geographic area, but the approach of analyzing phylogenetic patterns together with floristic or biogeographical relationships of the endemic taxa of an area, delimited by clear geomorphological features, could reveal evolutionary trends shaping the area.

## Abstract (es)

Se presenta una lista comentada de todas las especies endémicas conocidas de las Sierras de Córdoba y San Luis, una región montañosa aislada del centro de Argentina. A fin de obtener una mejor comprensión de la historia evolutiva, relaciones y edad de la flora regional, recopilamos información básica sobre afinidades florísiticas y biogeográficas de las endémicas, y relevamos su inclusión en estudios filogenéticos moleculares. El listado incluye 89 taxones (69 species y 20 taxones infraespecíficos), pertenecientes a 53 géneros y 29 familias. La distribución altitudinal de los endemismos no es uniforme, ya que el piso de vegetación inferior tiene más taxones endémicos que los pisos intermedio y superior. Treinta y dos géneros (60.3%) han sido incluidos en algún análisis filogenético, pero sólo 10 de ellos (18.8%) incluyeron taxones locales. Un total de 28 taxones endémicos de las Sierras CSL tienen una clara relación con una especie de amplia distribución del mismo género, o con una de distribución cercana al área. Las filogenias disponibles para algunos taxones muestran tiempos de divergencia entre 7.0 – 1.8 Ma; todos los endemismos del área son probablemente neoendemismos *sensu* Stebbins y Major. Aunque nuestro análisis estaba dirigido específicamente a un área geográfica particular, el enfoque de analizar patrones filogenéticos junto con relaciones florísticas y biogeográficas de los endemismos de un área delimitada por características geomorfológicas, podría revelar las tendencias evolutivas que modelaron el área.

## Introduction

### Why are endemic taxa important?

‘*The study and precise interpretation of the endemism of a territory constitute the supreme criterion, indispensable for arriving at any conclusions regarding the origin and age of its plant population. It enables us better to understand the past and the transformations that have taken place. It also provides us with a means of evaluating the extent of these transformations, the approximate epoch when they occurred, and the effects which they produced on the development of the flora and the vegetation*’ (Braun-Blanquet 1923: 223). Although many studies have dealt with the origin, classification and biology of endemism (e.g. Stebbins and Major 1965, Kruckeberg and Rabinowitz 1985, Hobhom 2013), this simple sentence by Josias Braun-Blanquet (1884–1980) illustrates well how some basic good definitions last through time. The study of plant endemism is important because it could improve our knowledge of the flora of a region in at least two different respects, which are briefly discussed below.

### Biogeography and evolution

The first aspect, perhaps the most traditional, has to do with biogeography and evolution of plants. The work of Stebbins and Major (1965) on the endemics of California outlined the basic elements to analyze when dealing with the endemic flora of a region: a) the floristic affinities and distribution of the endemics; b) the relationships of the endemic species with congeners (particularly for widely distributed taxa); c) the availability of a fossil record; and d) the use of genetic data to differentiate *paleo*- from *neo*-endemism.

These two concepts, paleo and neoendemic (Stebbins and Major 1965) apply to: a) ancient vestiges of taxa that were once more widespread, with their present distribution being a relict resulting of the reduction of their original habitats over time (paleoendemics); and b) relatively young species have only recently diverged from a parental entity, usually a widespread species (neoendemics).

The concepts of floristic affinities and fossil record availability have still more or less the same meaning as in the 1960’s, but today genetic data often provides a phylogenetic or phylogeographic context; these disciplines have matured into essential tools to understand evolutionary processes.

Biogeography counts the study of endemics and its distribution as one of its main subjects, since the existence of endemic taxa is related to geographic areas (Crisp et al. 2001). Both endemic taxa and restricted geographic areas are part of the same concept – i.e. taxa are considered endemic when they occur in a restricted area (Anderson 1994). Many studies have focused on the detection of areas of endemism (e.g. Myers et al. 2000, Crisp et al. 2001, Murray-Smith et al. 2009); a substantial number of endemic species in a geographical region often correlates with age and isolation of the area as these factors influence both the evolution (the formation and development of new taxa) and survival (the permanence of endemic relicts) (Lesica et al. 2006).

## Conservation

How should policy makers set priorities for conservation? Narrow endemic taxa often have priority in setting conservation policies (Chaplin et al. 2000) because narrow endemic plants are by definition rare, and in consequence face higher extinction risk due to environmental change (Crisp et al. 2001). Although there is controversy about what should be conserved, areas with high numbers of endemic species (hot spots) are often a preferred object of conservation policies and strategies because they offer the best reward for investment in conservation (Myers et al. 2000, Lamoreux et al. 2006, Ferreira and Boldrini 2011). But while Myers et al. (2000) defined 25 major biodiversity hotspots, and some have been well studied, e.g. the Brazilian Atlantic forest (Tabarelli et al. 1999, Morellato and Haddad 2000), there is still very little information on areas other than these 25 ‘major’ biodiversity hotspots, even though these are areas with fewer, but still a substantial number of, endemic species.

**Figure 1.**
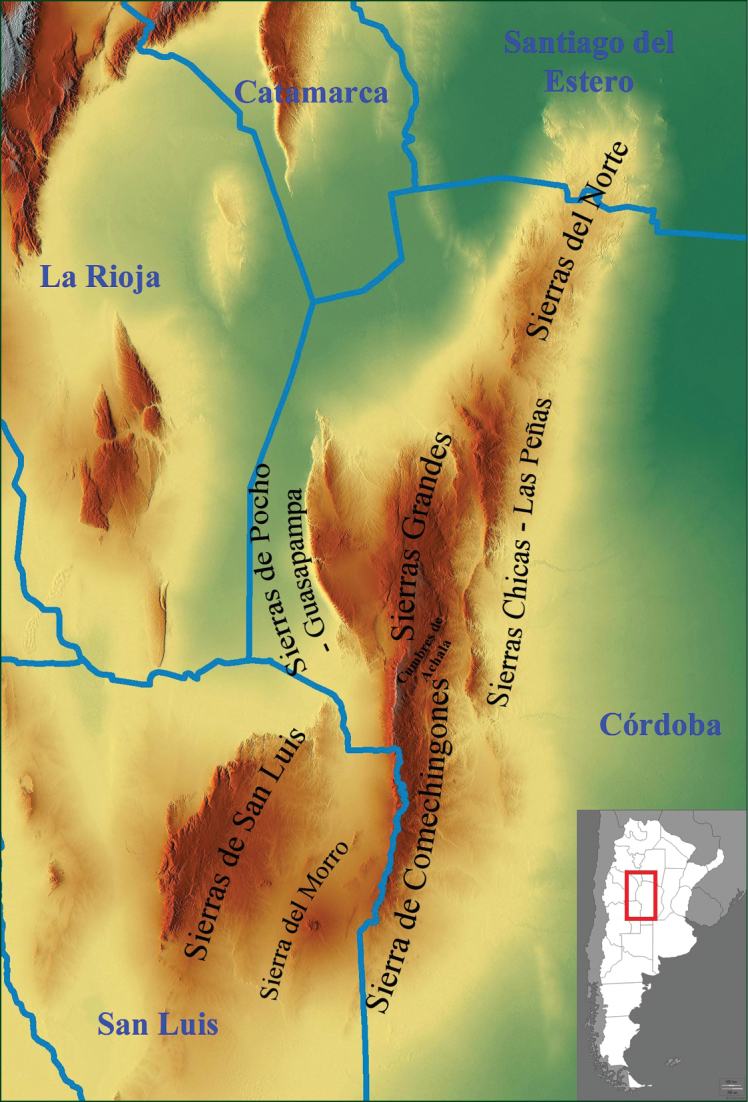
Map of the Sierras of Córdoba and San Luis (Sierras CSL).

Among all biotas, mountainous regions are especially rich in plant endemic species with restricted distribution, since those areas represent discontinuities in soil conditions and topography that promote differentiation in plant populations (Kruckeberg and Rabinowitz 1985; Lesica et al. 2006). The Sierras of Córdoba and San Luis (“Sierras CSL”) represents such an area, extending ca. 550 km in NE-SW length and about 110 km width, with the highest point represented by the Cerro Champaqui (2790 m). Sierras CSL are located in the center of Argentina, between 29° and 33°S, mostly in Córdoba and San Luis Provinces, except for a small northern portion extending into the neighboring province of Santiago del Estero (Fig. 1). With an overall northeast-southwest orientation and composition of Precambrian metamorphic blocks, the Sierras CSL are older than the Andes; they rise above Pampa plains of Quaternary origin (Baldo et al. 1996), and comprise six main sections (from north to south): *Sierras del Norte*, *Sierras Chicas-Las Peñas*, *Sierras Grandes-Sierra de Comechingones*, *Sierras de Pocho-Guasapampa*, *Sierra de San Luis* and *Sierra del Morro* (Fig. 1) (Carignano 1999).

**Figure 2.**
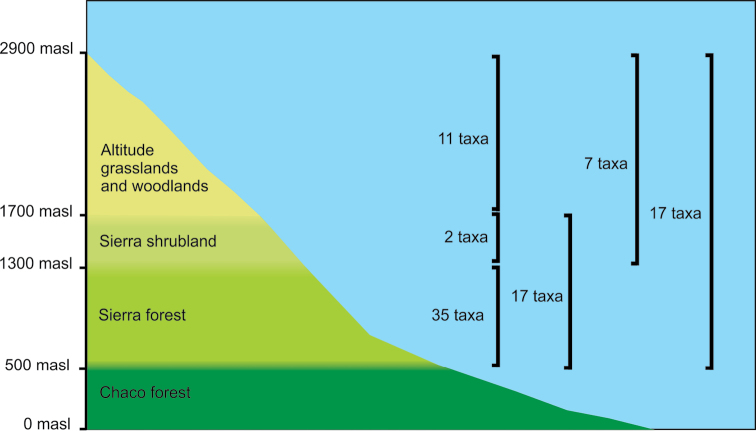
Vegetation belts in Sierras CSL.

Biogeographically, the flora of the Sierras CSL belongs to the Chaco Province of the Chacoan subregion (Morrone 2006); this is mainly xerophytic forest with shrubs and trees up to 15 m high (Cabrera and Willink 1980; Prado 1993a, b; Giorgis et al. 2011). Luti et al. (1979) described three main altitudinal vegetation belts for the Sierras CSL: the sierra forest, between 500 and 1300 meters above sea level; the sierra shrubland, between 1300 and 1700 meters; and finally, the altitude grasslands and woodlands, from 1700 meters upwards (Fig. 2). The upper belt is floristically different from the other two and shows affinities with Andean and Patagonian floristic elements (Cabido et al. 1998; Prado 1993a) and contains several endemics restricted to this altitude (Cabido et al. 1998). Of the three vegetation belts, the lower is the most exposed to anthropogenic threats because it lies close to the second largest city of Argentina (Córdoba); the attractive landscapes of the Sierras are also a preferred holiday destination in the country. Additional anthropogenic disturbances include fires and livestock grazing (Cingolani et al. 2013).

**Figure 3.**
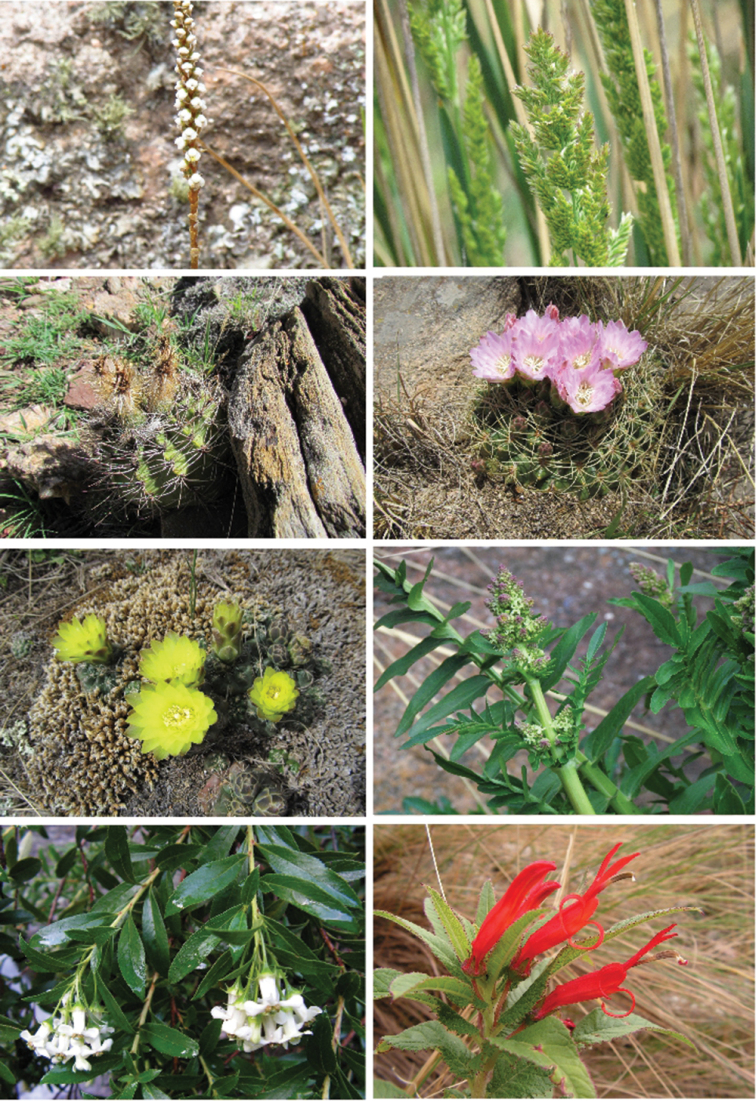
Representative endemic taxa of the Sierras CSL. (Clockwise) *Aa achalensis*, *Poa stuckertii*, *Acanthocalycium spiniflorum*, *Gymnocalycium monvillei*, *Gymnocalycium andreae*, *Valeriana ferax*, *Escallonia cordobensis*, Siphocampylus foliosus var. glabratus.

The implementation of conservation strategies needs in the first case basic information on the taxa object of potential conservation. Since previous works hinted at many endemic taxa present in the Sierras CSL (Cabido et al. 1998, Cantero et al. 2011, Oggero and Arana 2012), but specific evaluation of the endemic taxon richness of the Sierras CSL has not been done, we compiled a critical list of all species and infraspecific taxa endemic to the region. We then assessed the inclusion of the listed endemic taxa in molecular phylogenetic studies, as a means to estimate the evolutionary history of each studied taxon, specifically verifying relationships and divergence times (when available).

## Methods

We compiled a list using online resources, in particular Zuloaga et al. (2008) (updated to December 2014; http://www2.darwin.edu.ar/Proyectos/FloraArgentina/FA.asp) and the database of endemic plants of Argentina (http://www.lista-planear.org). We verified both the endemic status and the distribution of each taxon restricted to the Sierras CSL as defined by a cut-off altitude limit of 200 m. (i.e. endemic taxa from Córdoba and/or San Luis provinces found below this elevational limit were excluded from the list). Verification of taxa also included checking the validity of names and common synonyms; since estimates of biodiversity relies upon counting species names, including synonyms or *nomina dubia* would affect estimates of endemism (Alroy 2002). After this validation, we searched for information for each taxon regarding: 1) distribution, including altitudinal range; 2) life-form; 3) number of species in the genus; 5) inclusion in a molecular phylogenetic study; and 6) relationship to a widespread taxon of the same genus.

## Results

Of the relevant elements for studying endemism recognized by Stebbins and Major (1965), only the floristics of the Sierras CSL has been well studied (Cabido et al. 1987, 1998; Giorgis et al. 2011 and references therein), while the currently known fossil record is too sparse to be useful for studies of current vegetation (Leguizamon 1972, Balarino and Gutierrez 2006). We list 89 taxa (69 species and 20 infraspecific taxa, belonging to 53 genera and 29 families), which are found only in the provinces of Córdoba and San Luis at elevations above 200 m. Distribution, elevation and life form of each taxon are summarized in Table 1. The genus with the most endemics is *Gymnocalycium*, with 16 taxa. *Aristida*, *Gomphrena*, *Hieracium*, *Nassella*, *Portulaca*, *Siphocampylus*, *Senecio* and *Solanum* have 3 endemic taxa; *Grindelia*, *Hysterionica*, *Nothoscordum*, *Poa*, and *Valeriana* have 2 endemic taxa and the remaining genera each have one taxon.

**Table 1.** List of endemic species and infraspecific taxa of the Sierras of Córdoba and San Luis. *Distribution by Province* **D:** Córdoba: 1; San Luis: 2; Santiago del Estero: 3. *Life Form* **LF:** A-annual herb; P-perennial herb; S-shrub; SL-shrublet; V-perennial vine; SU-succulent, E-epiphytic.

	Family	Species	D	Elevation	LF
1	Alliaceae	*Nothoscordum achalense* Ravenna	1	1000–1800	P
2	Amaranthaceae	*Alternanthera pumila* O Stützer	1	1000–2000	P
3	Amaranthaceae	Gomphrena colosacana Hunz. & Subils var. andersonii Subils & Hunz.	2	500–1000	SL
4	Amaranthaceae	Gomphrena pulchella Mart. subsp rosea (Griseb.) Pedersen	1,2	500–1000	P
5	Amaranthaceae	Gomphrena pulchella Mart. var. bonariensis (Moq.) Pedersen	2	0 - 500	P
6	Amaryllidaceae	*Habranthus sanavirone* Roitman, A. Castillo, G. Tourn. & Uria	1	700–900	P
7	Amaryllidaceae	*Zephyranthes longistyla* Pax	1, 2, 3	1000–1500	P
8	Apiaceae	*Eryngium agavifolium* Griseb.	1, 2, 3	500–1000	P
9	Asteraceae	Grindelia cabrerae Ariza var alatocarpa Ariza	1	0–500	SL
10	Asteraceae	*Grindelia globularifolia* Griseb.	1	2000–2200	SL
11	Asteraceae	*Helenium argentinum* Ariza	1, 2, 3	200–1000	P
12	Asteraceae	*Hieracium achalense* Sleumer	1, 2	1000–2200	P
13	Asteraceae	*Hieracium cordobense* Sleumer	1, 2	1000–2000	P
14	Asteraceae	*Hieracium criniceps* Sleumer	1	1500–3000	P
15	Asteraceae	*Hypochaeris caespitosa* Cabrera	1, 2	1000–2500	P
16	Asteraceae	Hysterionica dianthifolia (Griseb.) Cabrera var dianthifolia	1	2000–3000	SL
17	Asteraceae	Hysterionica dianthifolia (Griseb.) Cabrera var pulvinata (Cabrera) Ariza	1	2000–2500	SL
18	Asteraceae	*Isostigma cordobense* Cabrera	1	500–1000	SL
19	Asteraceae	Mutisia castellanosii Cabrera var comechingoana Ariza	1	0–500	V
20	Asteraceae	*Senecio achalensis* Cabrera	1	1700–2800	SL
21	Asteraceae	*Senecio fragantissimus* Tortosa & A.Bartoli	2	800	S
22	Asteraceae	*Senecio retanensis* Cabrera	1, 2	2200–2800	SL
23	Asteraceae	*Soliva triniifolia* Griseb.	1		A
24	Asteraceae	*Trichocline plicata* Hook. & Arn.	1, 2	1000–3000	P
25	Berberidaceae	*Berberis hieronymi* C.K.Schneid	1	1000–2000	S
26	Brassicaceae	*Mostacillastrum carolinense* (Scappini, C.A.Bianco & Prina) Al-Shehbaz	2	1500–1700	SL
27	Bromeliaceae	Tillandsia xiphioides Ker Gawl. var. minor L.Hrom.	1, 2	1000–1500	E
28	Cactaceae	*Acanthocalycium spiniflorum* (K Schum) Backeb.	1, 2	1000–1500	SU
29	Cactaceae	*Gymnocalycium achirasense* H.Till & Schatzl ex H.Till	1, 2	500–1000	SU
30	Cactaceae	*Gymnocalycium andreae* (Boed) Backeb	1	1500–2500	SU
31	Cactaceae	*Gymnocalycium bruchii* (Speg) Hosseus	1, 2	1000–2000	SU
32	Cactaceae	*Gymnocalycium calochlorum* (Boed) Y.Itô	1	500–1500	SU
33	Cactaceae	*Gymnocalycium capillense* (Schick) Hosseus	1	500–1500	SU
34	Cactaceae	*Gymnocalycium carolinense* (Neuhuber) Neuhuber	2	1500–2000	SU
35	Cactaceae	Gymnocalycium castellanosii Backeb. subsp. ferocius (H.Till & Amerhauser) Charles	1	500–700	SU
36	Cactaceae	*Gymnocalycium erinaceum* J.G.Lamb.	1	500–1500	SU
37	Cactaceae	Gymnocalycium gibbosum (Haworth) Pfeiffer ex Mittler subsp. borthii (Koop ex H.Till) Charles	2	500–800	SU
38	Cactaceae	*Gymnocalycium horridispinum* Frank ex H.Till	1	500–700	SU
39	Cactaceae	*Gymnocalycium monvillei* (Lem) Britton & Rose	1, 2	500–2000	SU
40	Cactaceae	Gymnocalycium mostii (Gürke) Britton & Rose subsp. mostii	1	500–1000	SU
41	Cactaceae	Gymnocalycium mostii (Gürke) Britton & Rose subsp. valnicekianum (Jajó) Meregalli & Charles	1	500–1000	SU
42	Cactaceae	*Gymnocalycium neuhuberi* H.Till & W.Till	2	500–1500	SU
43	Cactaceae	*Gymnocalycium quehlianum* (F Haage ex Quehl) Vaupel ex Hosseus	1	500–1000	SU
44	Cactaceae	*Gymnocalycium robustum* R Kiesling, O.Ferrari & Metzing	1	0–500	SU
45	Campanulaceae	Siphocampylus foliosus Griseb. var. glabratus E.Wimm	1	1000–1500	SL
46	Campanulaceae	Siphocampylus foliosus Griseb. var. minor Zahlbr.	1	500–1500	SL
47	Campanulaceae	*Siphocampylus lorentzii* E.Wimm.	1	500–1500	SL
48	Caryophyllaceae	*Cerastium argentinum* (Pax) F.N.Williams	1		P
49	Cyperaceae	*Carex monodynama* (Griseb.) G.A.Wheeler	1	2600–2900	P
50	Escalloniaceae	*Escallonia cordobensis* (Kuntze) Hosseus	1, 2	1000–2500	S
51	Fabaceae	Adesmia cordobensis var appendiculata Ulibarri & Burkart	2	900–1100	SL
52	Fabaceae	*Apurimacia dolichocarpa* (Griseb.) Burkart	1	1800–3000	S
53	Fabaceae	*Astragalus parodii* I.M.Johnst.	1	1000–2500	P
54	Fabaceae	*Mimosa cordobensis* Ariza	1	0–500	S
55	Fabaceae	*Prosopis campestris* Griseb.	1, 2	500–2000	S
56	Fabaceae	*Sophora linearifolia* Griseb.	1, 2	1000–1500	SL
57	Gencianaceae	*Gentianella parviflora* (Griseb) T.N.Ho	1	1500–2500	A
58	Geraniaceae	*Geranium parodii* I.M.Johnst.	1, 2	1800–2600	P
59	Iridaceae	*Calydorea undulata* Ravenna	1	800–1000	P
60	Loasaceae	*Blumenbachia hieronymi* Urb.	1, 2	1900–2500	A
61	Malvaceae	*Sphaeralcea cordobensis* Krapov.	1, 2, 3	500–1000	SL
62	Orchidaceae	*Aa achalensis* Schltr.	1, 2	1500–2500	P
63	Plantaginaceae	*Plantago densa* (Pilg.) Rahn	1, 2	100–1800	P
64	Poaceae	Aristida minutiflora Caro var. glabriflora Caro	1, 2	500–1000	P
65	Poaceae	*Aristida multiramea* Hack.	1, 2	0–1000	P
66	Poaceae	*Aristida sayapensis* Caro	2	500–1000	P
67	Poaceae	*Cenchrus rigidus* (Griseb.) Morrone	1, 2	100–800	P
68	Poacaeae	*Danthonia melanathera* (Hack.) Bernardello	1, 2	1200	P
69	Poacaeae	*Melica decipiens* Caro	1, 2	1500–200	P
70	Poaceae	*Nassella hunzikeri* (Caro) Barkworth	1, 2	900–1500	P
71	Poaceae	*Nassella nidulans* (Mez.) Barkworth	1, 2	500–1500	P
72	Poaceae	*Nassella stuckertii* (Hack.) Barkworth	1	500–1500	P
73	Poaceae	*Poa hubbardiana* Parodi	1, 2	1400–2100	P
74	Poaceae	*Poa stuckertii* (Hack.) Parodi	1, 2	500–1500	P
75	Poaceae	Trichloris pluriflora E. Fourn. f. macra Hack.	1	500–1100	P
76	Poaceae	*Tridens nicorae* Anton	1, 2	1500	P
77	Portulacaceae	Portulaca confertifolia Hauman var. cordobensis D.Legrand	1, 2	500–1000	P
78	Portulacaceae	Portulaca obtusifolia D. Legrand var. obtusifolia	1	0–500	P
79	Portulacaceae	*Portulaca ragonesei* D.Legrand	1	200–400	P
80	Rosaceae	*Geum brevicarpellatum* F.Bolle	1	500–1500	P
81	Rubiaceae	Borreria eryngioides Cham & Schltdl. var. ostenii (Standl.) E.L.Cabral & Bacigalupo	1, 2	500–1000	P-SL
82	Rubiaceae	*Richardia coldenioides* Rusby	1	2700	P
83	Solanaceae	*Solanum concarense* Hunz.	2	500–1000	P
84	Solanaceae	*Solanum ratum* C.V.Morton	1	0–1000	P
85	Solanaceae	*Solanum restrictum* C.V.Morton	1	500–1500	P
86	Valerianaceae	*Valeriana ferax* (Griseb) Höck	1	2100–2300	P
87	Valerianaceae	*Valeriana stuckertii* Briq.	1, 2	1000–2500	P
88	Verbenaceae	Junellia bisulcata (Hayek) Moldenke var. campestris (Griseb.) Botta	1, 3	1000–2000	S
89	Verbenaceae	*Parodianthus capillaris* Tronc.	1	0–500	S

### Checklist of the endemic taxa of the Sierras of Córdoba and San Luis

All vouchers listed are from Argentina. Province (Córdoba, San Luis or Santiago de Estero) and Departamento (Depto.) are detailed for each where data are available.

ALLIACEAE

***Nothoscordum achalense*** Ravenna, Onira 3: 1. 1990.

Voucher: *Hunziker, A. T. 12919*, Prov. Córdoba, Depto. San Alberto, Sierra Grande, Pampa de Achala, en las inmediaciones de Monolito, 31°41'29"S, 65°6'5"W, (CORD)

AMARANTHACEAE

***Alternanthera pumila*** O. Stützer, Repert. Spec. Nov. Regni Veg. Beih. 88: 45. 1935.

Syn.: Alternanthera pumila O. Stützer var. coarctata O. Stützer.

Voucher: *Cantero, J. J. 6315*, Prov. Córdoba, Depto. Río Cuarto, Achiras (Monte Guazú), 33°2'36"S, 64°59'25"W, (CORD)

***Gomphrena colosacana*** Hunz. & Subils var. ***andersonii*** Subils & Hunz., Hickenia 1: 71, fig. 1A, B. 1977.

Voucher: *Chiapella, J. 1486*, Prov. San Luis, Depto. Belgrano, camino de acceso al Parque Nacional Sierra de Las Quijadas, a 3 km de la Ruta n° 147, antes de Hualtarán, 32°29'S, 67°0'60"W, (CORD)

***Gomphrena pulchella*** Mart var. ***bonariensis*** (Moq.) Pedersen, Darwiniana 20 (1–2): 292. 1976.

Voucher: *Vignati, M. A. 143*, Prov.San Luis, Depto. La Capital, (LP)

***Gomphrena pulchella*** Mart subsp. ***rosea*** (Griseb.) Pedersen, Darwiniana 20 (1–2): 292. 1976.

Syn.: Gomphrena perennis L. var. rosea Griseb.; *Gomphrena rosea* Griseb.

Voucher: *Nicora, E. G. 1858*, Prov. Córdoba, Depto. Colón, (SI)

AMARYLLIDACEAE

***Habranthus sanavirone*** Roitman, J. A. Castillo, G. M. Tourn & Uria, Novon 17(3): 393, fig. 1. 2007.

Voucher: *Roitman, G. s.n*, Prov. Córdoba, Depto. Cruz del Eje, San Marcos Sierras, (BAA)

***Zephyranthes longistyla*** Pax, Bot. Jahrb. Syst. 11: 323. 1891.

Voucher: Romanutti, A. 198, Prov. Córdoba, Depto. Punilla, Quebrada del Condorito, en el sendero hacia la Quebrada, 31°37'34"S, 64°42'22"W, (CORD)

APIACEAE

***Eryngium agavifolium*** Griseb., Abh. Königl. Ges. Wiss. Göttingen 19: 155. 1874.

Voucher: *Ariza Espinar, L. 3222*, Prov. Córdoba, Depto. Punilla, camino a las Altas Cumbres, yendo hacia El Cóndor, unos 6 km después de Puesto Pedernera, (CORD)

ASTERACEAE

***Grindelia cabrerae*** Ariza var. ***alatocarpa*** Ariza, Kurtziana 20: 170. 1989.

Voucher: *Chiarini, F. 1049*, Córdoba, Depto., San Justo, 30°56'22"S, 62°53'1"W, (CORD)

***Grindelia globularifolia*** Griseb., Symb. Fl. Argent. 178. 1879.

Voucher: *Cerana, M. M. 1806*, Prov. Córdoba, Depto. Punilla, Los Gigantes, 31°11'55"S, 64°35'1"W, (CORD)

***Helenium argentinum*** Ariza, Phytochemistry 31(5): 1626. 1992.

Voucher: *Cantero, J. J. 5618*, Córdoba, Depto. Río Cuarto, El Cóndor, 31°7'55"S, 64°46'47"W, (CORD)

***Hieracium achalense*** Sleumer, Bot. Jahrb. Syst. 77(1): 121. 1956.

Voucher: *Cerana, M. M. 1660*, Prov. Córdoba, Depto. Punilla, Cerro Uritorco, 31°11'55"S, 64° 35'1"W, (CORD)

***Hieracium cordobense*** Sleumer, Bot. Jahrb. Syst. 77(1): 120. 1956.

Syn.: Hieracium cordobense Sleumer var. mollisetum Sleumer.

Voucher: *Cerana, M. M.1662*, Prov. Córdoba, Depto. Punilla, Cerro Uritorco, Cima, 31°11'55"S, 64°35'1"W, (CORD)

***Hieracium criniceps*** Sleumer, Bot. Jahrb. Syst. 77(1): 116. 1956.

Syn.: *Hieracium petrophyes* Sleumer.

Voucher: *Hunziker, A. T. 11446*, Prov. Córdoba, Depto. Punilla, Sierra Grande (falda este), cuesta de Copina, entre Copina y Pampa de Achala, 31°34'31"S, 64°39'45"W, (CORD)

***Hypochaeris caespitosa*** Cabrera, Darwiniana 9: 376. 1951.

Voucher: *Cantero, J. J. 5596*, Prov. Córdoba, Depto. Río Cuarto, El Pantano (mármoles), 31°12'4"S, 64°48'20"W, (CORD)

***Hysterionica dianthifolia*** (Griseb.) Cabrera var. ***dianthifolia***, Notas Mus. La Plata, Bot. 11(53): 352. 1946.

Voucher: *Hunziker, A. T. 9649*, Prov. Córdoba, Depto. Calamuchita, Sierra de Comechingones (falda este), Cumbre de Cerro Champaquí, 31°59'15"S, 64°56'14"W, (CORD)

***Hysterionica dianthifolia*** (Griseb.) Cabrera var. ***pulvinata*** (Cabrera) Ariza, Darwiniana 22(4): 540. 1980.

Syn.: *Hysterionica pulvinata* Cabrera; *Neja pulvinata* (Cabrera) G.L.Nesom.

Voucher: *Ariza Espinar, L. 3461*, Prov. Córdoba, Depto. San Alberto, Pampa de Achala, entre camino Altas Cumbres y el Colegio del Padre Liqueno, (CORD)

***Isostigma cordobense*** Cabrera, Notas Mus. La Plata, Bot. 19(22): 202, f. 5. 1959.

Syn.: Isostigma crithmifolium Less. var. nanum Sherff.

Voucher: *Cantero, J. J. 5488*, Prov. Córdoba, Depto. Río Cuarto, Árbol Seco (serpentitas), 32°12'26"S, 64°41'40"W, (CORD)

***Mutisia castellanosii*** Cabrera var. ***comechingoniana*** Ariza, Bol. Soc. Argent. Bot. 35: 173. 2000.

Voucher: *Ariza Espinar, L. 3217*, Prov. Córdoba, Depto. Punilla, Sierra Chica (falda oeste), Los Terrones, 31°11'55"S, 64°35'01"W, (CORD)

***Senecio achalensis*** Cabrera, Notas Mus. La Plata, Bot. 1(4): 92. 1935.

Voucher: *Hunziker, A. T. 18048*, Prov. Córdoba, Depto. Punilla, Sierra Chica, Cerro Uritorco, falda occidental, 30°50'45"S, 64°28'12"W, (CORD)

***Senecio fragantissimus*** Tortosa & A.Bartoli, Novon 15(4): 646. 2005.

Voucher: *Covas, G. 1337*, Prov. San Luis, (LP)

***Senecio retanensis*** Cabrera, Notas Mus. La Plata, Bot. 4(21): 100. 1939.

Syn.: Senecio sectilis Griseb. var. radiatus Griseb.

Voucher: *Hunziker, A. T. 9641*, Prov. Córdoba, Depto. Calamuchita, Sierra de Comechingones (Falda este): En la falda oriental del Cerro Champaquí, 32°11'4"S, 64°37'1"W, (CORD)

***Soliva triniifolia*** Griseb., Abh. Königl. Ges. Wiss. Göttingen 24: 202. 1879.

Voucher: *Cabido, M. 6865*, Prov. Córdoba, Depto. San Alberto, Sierra Grande, Pampa de Achala, en la Estancia San Alejo, (CORD)

***Trichocline plicata*** D. Don ex Hook. & Arn., Comp. Bot. Mag. 1: 103. 1835.

Voucher: *Cantero, J. J. 5903*, Prov. Córdoba, Depto. Colón, Candonga, 31°4'30"S, 64°20'16"W, (CORD)

BERBERIDACEAE

***Berberis hieronymi*** C.K.Schneid., Bull. Herb. Boissier, sér. 2, 5: 394. 1905.

Syn.: Berberis ruscifolia Lam. var. subintegrifolia Kurtz.

Voucher: *Romanutti, A. 212*, Prov. Córdoba, Depto. Punilla, Quebrada del Condorito, en el sendero hacia la Quebrada, 31°37'34"S, 64°42'22"W, (CORD)

BRASSICACEAE

***Mostacillastrum carolinense*** (Scappini, C.A.Bianco & Prina) Al-Shehbaz, Darwiniana 44(2): 346. 2006.

Syn.: *Sysimbrium carolinense* Scappini, C.A. Bianco & Prina.

Voucher: *Scappini, E. G. 5316*, Prov. San Luis, (RIOC)

BROMELIACEAE

***Tillandsia xiphioides*** Ker Gawl. var. ***minor*** L.Hrom., Die Bromelie 3:61–65. 1989.

Voucher: *Zavala-Gallo, L. s.n.* (SI 96882), Prov. San Luis, Depto. Belgrano, Sierra de Las Quijadas, 32°45'9"S, 66°44'49"W, (SI)

CACTACEAE

***Acanthocalycium spiniflorum*** (K.Schum.) Backeb., Kaktus-ABC [Backeb. & Knuth] 226. 1936.

Syn.: *Echinopsis spiniflora* K.Schum., *Lobivia spiniflora* (K.Schum) Britton & Rose

Voucher: *Schlumpberger, B. O. 323*, Prov. Córdoba, Depto. Minas, Agua de Ramón, (CORD)

***Gymnocalycium achirasense*** H.Till & Schatzl ex H.Till, Kakteen Sukk. 38(8): 191. 1987.

Syn.: Gymnocalycium monvillei (Lem.) Britton & Rose subsp. achirasense (H.Till & Schatzl ex H.Till) H.Till; *Gymnocalycium horridispinum* Frank ex H.Till var. *achirasense* (H.Till & Schatzl ex H. Till) Lodé; Gymnocalycium horridispinum subsp. achirasense (H.Till & Schatzl ex H.Till) Charles

Voucher: *Demaio, P. 489*, Prov. Córdoba, Depto. Río Cuarto, Alpa Corral (CORD)

***Gymnocalycium andreae*** (Boed.) Backeb. Kaktus-ABC [Backeb. & Knuth]: 285. 1935.

Syn.: Gymnocalycium andreae (Boed.) Backeb. f. svecianum Pazout ex H.Till; Gymnocalycium andreae (Bödeker) Backeb. subsp. maznetteri Rausch; Gymnocalycium andreae (Boed.) Backeb. var. fechseri H.Till.

Voucher: *Demaio, P. 480*, Prov. San Luis, Depto. Junín, cuesta de Merlo, (CORD)

***Gymnocalycium bruchii*** (Speg.) Hosseus, Revista Centro Estud. Farm. Córdoba 2(6): 22. 1926.,

Syn.: *Gymnocalycium lafaldense* Vaupel; *Gymnocalycium albispinum* Backeb.; Gymnocalycium andreae (Boed.) Backeb. var. grandiflorum Krainz & Andreae; Gymnocalycium bruchii (Speg.) Hosseus var. brigittae Piltz; Gymnocalycium bruchii (Speg.) Hosseus var. niveum Rausch.

Voucher: *Demaio, P. 111*, Prov. Córdoba, Depto. Colón, Ruta Provincial E-66 (Camino del Pungo), 30°56'36"S, 64°23'16"W, (CORD)

***Gymnocalycium calochlorum*** (Boed.) Y.Itô, Cacti 1952: 90. 1952.

Syn.: *Gymnocalycium proliferum* (Backeb.) Backeb.; *Gymnocalycium amoenum* (H.Till) Lambert.

Voucher: *Kiesling, R. 9069*, Prov. Córdoba, Depto. San Alberto, Mina Clavero, (SI)

***Gymnocalycium capillaense*** (Schick) Hosseus, Revista Centro Estud. Farm. Córdoba 2(6): 16. 1926.

Syn.: *Gymnocalycium sigelianum* (Schick) Hosseus; *Gymnocalycium sutterianum* (Schick) Hosseus; *Gymnocalycium deeszianum* Dölz; *Gymnocalycium poeschlii* Neuhuber; *Gymnocalycium fischeri* Halda, Kupcák, Lukasik & Sladkovsky; *Gymnocalycium miltii* Halda, Kupcák, Lukasik & Sladkovsky; Gymnocalycium fischerii subsp. suyuquense Berger; *Gymnocalycium nataliae* Neuhuber.

Voucher: *Leuenberger, B. E.4389*, Córdoba, Depto. Punilla, 2 Km N of Capilla del Monte towards Charbonier, 30°51'S, 64°32'W, (CORD)

***Gymnocalycium carolinense*** (Neuhuber) Neuhuber, Gymnocalycium 18(4): 639–640. 2005.

Voucher: *Demaio, P. 475*, Prov. San Luis, Depto. Coronel Pringles, La Carolina, (CORD)

***Gymnocalycium castellanosii*** Backeb. subsp. ***ferocius*** (H.Till & Amerhauser) Charles, Cactaceae Systematics Initiatives 20: 18. 2005.

Syn.: Gymnocalycium mostii subsp. ferocior H.Till & Amerhauser.

Voucher: *Borth, H. s.n.*, Prov. Córdoba, Depto. Minas, Agua de Ramón, (CORD)

***Gymnocalycium erinaceum*** Lambert, Succulenta 64: 64–66. 1985.

Syn.: *Gymnocalycium amerhauseri* H.Till; *Gymnocalycium lukasikii* Halda & Kupcak; *Gymnocalycium papschii* H.Till; *Gymnocalycium gaponii* Neuhuber; *Gymnocalycium walteri* H.Till.

Voucher: *Demaio, P. 108*, Prov. Córdoba, Depto. Colón, Ruta Provincial E-66 (Camino del Pungo), pasando Tres Cascadas, 30°56'58"S, 64°19'57"W, (CORD)

***Gymnocalycium gibbosum*** (Haw.) Pfeiff. ex Mittler subsp. ***borthii*** (Koop ex H.Till) Charles, Cactaceae Systematics Initiatives 20: 18. 2005.

Syn.: *Gymnocalycium berchtii* Neuhuber; *Gymnocalycium borthii* Koop ex H.Till subsp. *nogolense* Neuhuber.

Voucher: *Demaio, P. H. 479*, Prov. San Luis, Junín, Los Chañares, (CORD)

***Gymnocalycium horridispinum*** Frank ex H.Till, Kakteen And. Sukk. 38(8): 191. 1987.

Syn.: Gymnocalycium monvillei (Lem.) Britton & Rose subsp. horridispinum (Frank ex H.Till) H.Till.

Voucher: *Fechser, H. s.n.*, Prov. Córdoba, SW Salsacate, (WU)

***Gymnocalycium monvillei*** (Lem.) Britton & Rose, Cactaceae [Britton & Rose] 3: 161. 1922.

Syn.: *Gymnocalycium multiflorum* (Hook.) Britton & Rose; *Gymnocalycium brachyanthum* (Gürke) Britton & Rose; *Gymnocalycium grandiflorum* Backeb.; *Gymnocalycium schuetzianum* H.Till & Schatzl.

Voucher: *Demaio, P. 112*, Prov. Córdoba, Depto. Colón, Ruta Provincial E-66 (Camino del Pungo), 30°56'34"S, 64°23'53"W, (CORD)

***Gymnocalycium mostii*** (Gürke) Britton & Rose subsp. ***mostii***, Addisonia 3: 5. 1918.

Syn.: *Gymnocalycium kurtzianum* (Gürke) Britton & Rose.

Voucher: *Leuenberger, B. E. 4490*, Prov. Córdoba, Depto. Colón, 15–16 km W of Ascochinga on road to La Cumbre, (CORD)

***Gymnocalycium mostii*** (Gürke) Britton & Rose subsp. ***valnicekianum*** (Jajó) Meregalli & Charles, Cactaceae Systematics Initiatives 24. 2008.

Syn.: *Gymnocalycium inmemoratum* A. Castellanos & Lelong; *Gymnocalycium tobuschianum* Schick.; *Gymnocalycium prochazkianum* Sorma

Voucher: *Kiesling, R. 9069*, Prov. Córdoba, Depto. Punilla, Capilla del Monte, (SI)

***Gymnocalycium neuhuberi*** H.Till & W.Till, Gymnocalycium 5(1):59–60. 1992.

Voucher: *Demaio, P. H. 470*, Prov. San Luis, Depto. Belgrano, Suyuque, (CORD)

***Gymnocalycium quehlianum*** (F.Haage ex Quehl) Vaupel ex Hosseus, Revista Centro Estud. Farm. Córdoba 2(6): 22. 1926.

Syn.: Gymnocalycium quehlianum (F. Haage ex Quehl) Vaupel ex Hosseus var. rolfianum Schick.; Gymnocalycium quehlianum (F. Haage ex Quehl) Vaupel ex Hosseus var. zantnerianum Schick.; *Gymnocalycium stellatum* Speg.; Gymnocalycium stellatum Speg. var. flavispinum Bozsing ex H.Till & W.Till; Gymnocalycium stellatum Speg. var. kleinianum Rausch ex H.Till & W.Till.

Voucher: *Schlumpberger, B. O. 320*, Prov. Córdoba, Depto. Punilla, Capilla del Monte, El Cajón, (CORD)

***Gymnocalycium robustum*** R.Kiesling, O.Ferrari & Metzing, Cactus and Succulent Journal (US) 74(1): 4–8. 2002.

Syn.: *Gymnocalycium kuehhasii* Neuhuber & Sperling

Voucher: *Kiesling, R. 9883*, Prov. Córdoba, Depto. Ischilín, Quilino, 30°22'18"S, 64°39'31"W, (SI)

CAMPANULACEAE

***Siphocampylus foliosus*** Griseb. var. ***glabratus*** E.Wimm., Revista Sudamer. Bot. 2: 93. 1935.

Voucher: *Stuckert, T. J. V. 10536*, Prov. Córdoba, Depto. San Alberto, Mina Clavero, 31°41'29"S, 65°6'5"W, (CORD)

***Siphocampylus foliosus*** Griseb. var. ***minor*** Zahlbr., Revis. Gen. Pl. 3[3]: 189. 1898.

Voucher: *Stuckert, T. J. V. 10816*, Prov. Córdoba, (G)

***Siphocampylus lorentzii*** E.Wimm., Repert. Spec. Nov. Regni Veg. 29: 85. 1931.

Voucher: Lorentz, P. G. 697, Prov. Córdoba, (B)

CARYOPHYLLACEAE

***Cerastium argentinum*** (Pax) F.N.Williams, J. Bot. 36: 387. 1898.

Syn.: Cerastium nutans Raf. var. argentinum Pax

Voucher: *Hunziker, A. T. 6412*, Prov. Córdoba, Depto. San Alberto, Sierra Grande, Pampa de Achala, al costado del monolito (Ruta Prov. 14), 31°40'42"S, 64°50'11"W, (CORD)

CYPERACEAE

***Carex monodynama*** (Griseb.) G.A.Wheeler, Syst. Bot. 15: 656. 1990.

Syn.: Carex atropicta Steud. var. monodynama Griseb.; Carex atropicta Steud. var. pallescens Kurtz ex Kük.; Carex atropicta Steud. f. monodynama (Griseb.) Kük.; Carex atropicta Steud. f. pallescens (Kurtz ex Kük.) Kük.

Voucher: *Kurtz, F. 3080h*, Prov. Córdoba, Depto. Calamuchita, (CORD)

ESCALLONIACEAE

***Escallonia cordobensis*** (Kuntze) Hosseus, Bol. Acad. Nac. Ci. 26: 120–121, f. 18. 1921.

Syn.: Escallonia rubra (Ruiz & Pav.) Pers. var. cordobensis Kuntze; *Escallonia montana* auct. non Phil.

Voucher: *Ariza Espinar, L. 3494*, Prov. Córdoba, Depto. Punilla, Copina, 31°11'55"S, 64°35'1"W, (CORD)

FABACEAE

***Adesmia cordobensis*** Burkart var. ***appendiculata*** Ulibarri & Burkart, *Darwiniana* 38(1–2): 84. 2000.

Voucher: *Anderson, D. L. 1921*, Prov. San Luis, Depto. Pedernera, Cerro El Morro, Ea. La Guardia, (SI)

***Apurimacia dolichocarpa*** (Griseb.) Burkart, Physis (Buenos Aires) 20(58): 286. 1951.

Syn.: *Tephrosia dolichocarpa* Griseb.

Voucher: *Cabrera, A. L. 29655*, Prov. Córdoba, Depto. Pocho, Subida de Taninga, 31°21'30"S, 64°58'W, (SI)

***Astragalus parodii*** I.M.Johnst., J. Arnold Arbor. 28: 371. 1947.

Voucher: *Hieronymus, G. H. E. W. s.n*., Prov. Córdoba, Sierra de Achala, Cuesta del Gaucho, (CORD)

***Mimosa cordobensis*** Ariza, Lorentzia 6: 7–10, f. 1. 1986.

Voucher: *Ariza Espinar, L. 3014*, Prov. Córdoba, Depto. Totoral, El Sauce, 30°40'51"S, 63°55'25"W, (CORD)

***Prosopis campestris*** Griseb., Abh. Königl. Ges. Wiss. Göttingen 19: 132–133. 1874.

Voucher: *Lorentz, P. G. 2*, Prov. Córdoba, “Umgebung von Chañar, wenige Leguas nach Süd und Nord verschwindend”, (CORD)

***Sophora linearifolia*** Griseb., Symb. Fl. Argent. 110. 1879.

Voucher: *Hieronymus, G. H. E. W. 135*, Prov. Córdoba, orillas del río cerca del Molino de Ducas, (CORD)

GENTIANACEAE

***Gentianella parviflora*** (Griseb) T.N.Ho, Bull. Brit. Mus. (Nat. Hist.), Bot. 23(2): 63. 1993.

Voucher: *Ariza Espinar, L. 1390*, Prov. Córdoba, Depto. San Alberto, Pampa de Achala: Cerca de La Posta, 31°41'29"S, 65°6'5"W, (CORD)

GERANIACEAE

***Geranium parodii*** I.M.Johnst., Contr. Gray Herb. 81: 92. 1928.

Voucher: *Stuckert, T. 26029*, Prov. Córdoba, Depto. Cruz del Eje, Sierra de Achala, entre Tanti y Pampa de San Luis, 31°19'S, 64°35'W, (CORD)

IRIDACEAE

***Calydorea undulata*** Ravenna, Onira 6(1): 14. 2001.

Voucher: *Maldonado-Bruzzone, R. 1037*, Prov. Córdoba, Depto. Río Seco, Cerro Colorado, (LP)

MALVACEAE

***Sphaeralcea cordobensis*** Krapov., Lilloa 17: 214. 1949.

Voucher: *Cantero, J. J. 5388*, Prov. Córdoba, Depto. Calamuchita, Cañada de Alvarez, 32°22'1"S, 64°32'4"W, (CORD)

LOASACEAE

***Blumenbachia hieronymi*** Urb., Jahrb. Königl. Bot. Gart. Berlin 3: 249. 1884.

Voucher: *Hieronymus, G. H. E. W. 790*, Prov. Córdoba, Sierra de Achala, al pie del Cerro Champaquí, (CORD)

ORCHIDACEAE

***Aa achalensis*** Schltr., Repert. Spec. Nov. Regni Veg. 16: 358. 1920.

Voucher: *Ariza Espinar, L. 428*, Prov. Córdoba, Depto. Punilla, entre Cosquín y Parque Siquiman 31°11'55"S, 64°35'1"W, (CORD)

PLANTAGINACEAE

***Plantago densa*** (Pilg.) Rahn, Nord. J. Bot. 3(3): 336. 1983.

Voucher: *Hieronymus, G. H. E. W. 603*, Prov. Córdoba, (CORD, F)

POACEAE

***Aristida minutiflora*** Caro var. ***glabriflora*** Caro, Kurtziana 1: 154. 1961.

Voucher: *Hunziker, A. T. 22472*, Prov. Córdoba, Depto. Pocho, Sierra de Pocho: entre Arroyo Piedras Rosadas y Arroyo de las Águilas, 31°25'57"S, 65° 25'38"W, (CORD)

***Aristida multiramea*** Hack., Anales Mus. Nac. Buenos Aires 21: 67. 1911.

Syn.: Aristida adscensionis L. var. laevis Hack.

Voucher: *Hunziker, A. T. 14026*, Prov. Córdoba, Depto. Pocho, Sierra de Pocho (falda oeste): Ruta 20, cerca de los Túneles, 31°25'57"S, 65°25'38'W, (CORD)

***Aristida sayapensis*** Caro, *Kurtziana* 1: 159. 1961.

Voucher: *Anderson, D. L. 2202*, Prov. San Luis, Depto. General Pedernera, Ruta 148, 13 km al norte de Villa Mercedes, 34°1'20"S, 65°34'39'W, (CORD)

***Cenchrus rigidus*** (Griseb.) Morrone, Ann. Bot. (Oxford) 106: 129. 2010.

Syn.: *Pennisetum rigidum* (Griseb.) Hack., *Gymnotrix rigida* Griseb.

Voucher: *Stuckert, T. J. V. 18737*, Prov. Córdoba, Depto. Río Primero, Estancia San Teodoro, 31°1'24"S, 63°27'21"W, (CORD)

***Danthonia melanathera*** (Hack.) Bernardello, Kurtziana 10: 249. 1977.

Syn.: *Danthonia cirrata* Hack. & Arechav. var. *melanathera* Hack.

Voucher: *Krapovickas, A. 7414*, Prov. Córdoba, Depto. Punilla, entre Copina y la Pampa de Achala, 31°11'55"S, 64°35'1"W, (CORD)

***Melica decipiens*** Caro, Kurtziana 5: 288, fig. 5. 1969.

Syn.: Melica violacea Cav. var. glabrior Papp; Melica violacea Cav. f. mucronata Papp.

Voucher: *Hunziker, A. T. 9687*, Prov. Córdoba, Depto. San Javier, Sierra Grande, bajando del cerro Champaquí, 32°5'3"S, 65°6'5"W, (CORD)

***Nassella hunzikeri*** (Caro) Barkworth, Taxon 39(4): 610. 1990.

Syn.: *Stipa hunzikeri* Caro

Voucher: *Cantero, J. J. 5539*, Prov. Córdoba, Depto. Río Cuarto, Iguazú, 31°3'51"S, 64° 47'39"W, (CORD)

***Nassella nidulans*** (Mez.) Barkworth, Taxon 39(4): 611. 1990.

Syn.: *Stipa nidulans* Mez.

Voucher: *Hunziker, A. T. 18052*, Prov. Córdoba, Depto. Punilla, Sierra Chica, Falda Oeste del Cerro Uritorco, 31°11'55"S, 64°35'1"W, (CORD)

***Nassella stuckertii*** (Hack.) Barkworth, Taxon 39(4): 612. 1990.

Syn.: *Stipa stuckertii* Hack.

Voucher: *Hunziker, A. T. 8646*, Prov. Córdoba, Depto. Punilla, camino a Los Gigantes, El Vallecito, 31°11'55"S, 64°35'1"W, (CORD)

***Poa hubbardiana*** Parodi, Notas Mus. La Plata, Bot. 2: 10–13, f.4. 1937.

Voucher: *Hunziker, A. T. 8682*, Prov. Córdoba, Depto. Punilla, Sierra Grande, Cerro de La Cruz, al este de Los Gigantes, 31°11'55"S, 64°35'1"W, (CORD)

***Poa stuckertii*** (Hack.) Parodi, Physis (Buenos Aires) 11: 137. 1932.

Syn.: Poa lanigera Nees var. stuckertii Hack.

Voucher: *Hunziker, A. T. 9657*, Prov. Córdoba, Depto. Calamuchita, Sierra Grande, Cumbre del Cerro Champaquí, 32°11'4"S, 64° 37'1"W, (CORD)

***Trichloris pluriflora*** E. Fourn. f. ***macra*** Hack., Anales Mus. Nac. Buenos Aires ser. 3, 4: 116. 1904.

Voucher: *Hunziker, A. T. 14868*, Prov.San Luis, Depto. Junín, Sierra de San Luis, Quebrada del Tigre, entre Santa Rosa y Bañado de Cautana, 32°18'45"S, 65°16'39"W, (CORD)

***Tridens nicorae*** Anton, Kurtziana 10: 51, fig. 1977.

Syn.: *Antonella nicorae* (Anton) Caro.

Voucher: *Anderson, D. L. 1686*, Prov. San Luis, Depto. La Capital, Cerro El Lince, faldeo oriental, 33°43'47"S, 66°30'47"W, (CORD)

PORTULACACEAE

***Portulaca confertifolia*** Hauman var. ***cordobensis*** D. Legrand, Lilloa 17: 360, fig. 19. 1949.

Voucher: *Soriano, A. 791*, Prov. Córdoba, Salinas Grandes, km 907, (SI)

***Portulaca obtusifolia*** D. Legrand var. ***obtusifolia***, Comun. Bot. Mus. Hist. Nat. Montevideo 3(32): [1], tab. 1. 1959.

Voucher: *Sayago, M. 2311*, Prov. Córdoba, Depto. Río Seco, en el centro de Saladillo, Villa Candelaria, 29°58'45"S, 63°15'49"W, (CORD)

***Portulaca ragonesei*** D. Legrand, Lilloa 17: 333, tab. 2. 1949.

Voucher: *Ragonese, A. E. s.n.*, Prov. Córdoba, (BAB)

ROSACEAE

***Geum brevicarpellatum*** F.Bolle, Repert. Spec. Nov. Regni Veg. Beih. 72: 54. 1933.

Voucher: *Hieronymus, G. H. E. W. 35*, Prov. Córdoba, Depto. San Alberto, Sierra Achala, Quebrada del Chorro, al Este de Los Gigantes, (CORD)

RUBIACEAE

***Borreria eryngioides*** Cham. & Schltdl. var. ***ostenii*** (Standl.) E.L.Cabral & Bacigalupo, Opera Bot. Belg. 7: 317. 1996.

Syn.: *Borreria ostenii* Standl.

Voucher: *Ariza Espinar, L. 1260*, Prov. Córdoba, Depto. Capital, Barrio San Martín, 31°23'27"S, 64°11'27"W, (CORD)

***Richardia coldenioides*** Rusby, Mem. Torrey Bot. Club 4: 208. 1895.

Syn.: *Richardsonia coldenioides* (Rusby) Buchtien

Voucher: *Burkart, A. 10443*, Prov. Córdoba, Depto. Calamuchita, Río Tercero, 32°11'4"S, 64° 37'1"W, (SI)

SOLANACEAE

***Solanum concarense*** Hunz., Kurtziana 20: 190, fig. 2. 1989.

Voucher: *Hunziker, A. T. 14547*, Prov. San Luis, Depto. Chacabuco, cerca de Concarán, Santa Rosa, 32°42'38"S, 65°12'6"W, (CORD)

***Solanum ratum*** C.V.Morton, Revis. Argentine Sp. Solanum 130 (-132), figs. 121-L, 15. 1976.

Voucher: *Chiarini, F. 818*, Prov. Córdoba, Depto. Punilla, entre Cerro Blanco y La Ollada, El Durazno, 31°11'55"S, 64°35'1"W, (CORD)

***Solanum restrictum*** C.V.Morton, Revis. Argentine Sp. Solanum 128 (-130), figs. 12E-N, 14. 1976.

Voucher: *Chiarini, F. 794*, Prov. Córdoba, Depto. Punilla, La Cumbre, alrededores de la Estancia El Rosario, 31°59'2"S, 64°28'13"W, (CORD

VALERIANACEAE

***Valeriana ferax*** (Griseb.) Höck, Bot. Jahrb. Syst. 3(1): 55. 1882.

Syn.: *Phyllactis ferax* Griseb.

Voucher: *Hunziker, A. T. 9683*, Prov. Córdoba, Depto. San Javier, Sierra Grande, Cerro Champaquí, 32°5'3"S, 65°6'5"W, (CORD)

***Valeriana stuckertii*** Briq., Annuaire Conserv. Jard. Bot. Genève 20: 442. 1919.

Voucher: *Stuckert, T. J. V. 12063*, Prov. Córdoba, Depto. Santa María, Sierra Chica, 31°38'59"S, 64°15'28"W, (CORD)

VERBENACEAE

***Junellia bisulcata*** (Hayek) Moldenke var. ***campestris*** (Griseb.) Botta, Hickenia 2: 127. 1995.

Syn.: Junellia juniperina (Lag.) Moldenke var. campestris (Griseb.) Moldenke; Verbena juniperina Lag. var. campestris Griseb.

Voucher: *Krapovickas, A. 7751*, Prov. Córdoba, Depto. Pocho, Taninga, 31°25'57"S, 65°25'38"W, (SI)

***Parodianthus capillaris*** Tronc., Darwiniana 18: 21. 1973.

Voucher: *Hunziker, A. T. 24987*, Prov. Córdoba, Depto. Sobremonte, Sierra del Norte, 6 km al oeste de San Francisco del Chañar, hacia Lucio V. Mansilla, 29°44'12"S, 64°7'57"W, (CORD)

### Distribution of the endemic taxa

The altitudinal distribution of the endemic taxa in the Sierras CSL is shown in Table 2 and Fig. 2. There are *exclusive* taxa (i.e., present *only* in a single altitudinal belt) and also *shared* taxa (present in more than one altitudinal belt). Among the exclusive taxa, the lower Sierra forest belt has 35 taxa, the intermediate Sierra shrubland belt has 2 taxa and the upper grasslands and woodlands belt has 11 taxa. The presence of taxa in more than one belt is depicted in the last three columns of Table 2, that shows which taxa are present in which combination of belts; among the taxa which are present in two belts, the combination of lower and middle belts has 17 taxa and the combination of middle and upper belts has 7 taxa. Finally there are 17 taxa that are present in all the three belts.

**Table 2.** Summary of altitudinal distribution of endemic taxa in the Sierras CSL. Taxa identification numbers as in Table 1.

Vegetation belt or combination	lower	middle	upper	lower/middle	middle/upper	lower/middle/upper
taxa	35	2	11	17	7	17
percentage	39.32	2.29	12.35	19.54	8.04	19.54
taxa ID	3, 4, 5, 7, 8, 17, 18, 20, 28, 34, 36, 37, 39, 40, 41, 42, 47, 49, 52, 57, 61, 64, 65, 66, 67, 68, 75, 77, 78, 79, 81, 82, 83, 84, 89	25, 76	9, 15, 16, 19, 21, 22, 47, 50, 56, 60, 86	6, 10, 26, 27, 31, 32, 35, 43, 44, 45, 54, 70, 71, 72, 74, 80, 85	13, 29, 33, 55, 62, 69, 73	1, 2, 11, 12, 14, 23, 24, 30, 38, 48, 51, 53, 58, 59, 63, 87, 88

### Phylogenetic knowledge of the endemic taxa of the CSL Sierras

The inclusion of the endemic taxa of the Sierras CSL in phylogenetic studies has been minimal; from a total of 53 genera with endemic taxa present in the area, 32 (60.3%) have been included in at least one molecular phylogenetic analysis, but only 10 studies (18.8%) have a species endemic to the Sierras CSL: *Acanthocalycium*, *Blumenbachia*, *Eryngium*, *Escallonia*, *Grindelia*, *Gymnocalycium*, *Portulaca*, *Prosopis*, *Sphaeralcea* and *Tillandsia* (Table 3).

**Table 3.** Phylogenetic knowledge of the endemic taxa of the CSL Sierras.

Family	Genus	Phylogeny of the genus including endemic species of CSL Sierras
Amaranthaceae	*Gomphrena*	Moore et al. 2012
Apiaceae	*Eryngium*	Calviño et al. 2008
Bromeliaceae	*Tillandsia*	Barfuss et al. 2005
Cactaceae	*Acanthocalycium*	Schlumpberger and Renner 2012
Cactaceae	*Gymnocalycium*	Demaio et al. 2011
Escalloniaceae	*Escallonia*	Sede et al. 2013
Fabaceae	*Prosopis*	Catalano et al. 2007
Loasaceae	*Blumenbachia*	Hufford et al. 2005; Ackerman et al. 2006
Malvaceae	*Sphaeralcea*	Tate and Simpson 2003
Portulacaceae	*Portulaca*	Ocampo and Columbus 2012

### Assessment of the phylogenetic knowledge of the genera with endemic taxa of the Sierras CSL.

***Aa*.** South American genus with around 25 species, mostly in the Central and Northern Andes, and 5 species in Argentina (Luer 2008). The phylogeny by Álvarez-Molina and Cameron (2009) included some species of the genus *Aa*, but not the Sierras CSL endemic *Aa achalensis*. The genus was placed in the Altensteinia clade of the sub tribe Prescottiinae.

***Acanthocalycium*.** Endemic genus of the mountain ranges of Central Argentina, with 5 species (Kiesling 2008). Hunt (2006) synonymized this genus with *Echinopsis*. *Acanthocalycium spiniflorum* has been included in a molecular phylogeny (Schlumpberger and Renner 2012), which shows the genus as paraphyletic, embedded in *Echinopsis* sensu lato, in agreement with Hunt (2006). Regardless of the delimitation of the genus, *Acanthocalycium spiniflorum* is a long branch in the phylogram of Schlumpberger and Renner (2012). Arakaki et al. (2011) dated the diversification of the clade Trichocereinae (including *Acanthocalycium*) between 7.5–6.5 Ma. Hernández-Hernández et al. (2014) dated the divergence time of *Acanthocalycium spiniflorum* to ca. 2.5 Ma.

***Adesmia*.** This genus has ca. 240 species in South America, most of which are found in the Andes (Burkart 1967). In Argentina there are 198 species (Ulibarri 2008), and the Sierras CSL endemic Adesmia cordobensis var. appendiculata has not been included in a molecular phylogeny.

***Alternanthera*.** Cosmopolitan genus with ca. 100 species, mostly in tropical and warm regions of America. There are 36 species in Argentina (Borsch 2008a). A recent molecular study by Sanchez del Pino et al. (2012) included other Argentinean species, such as *Alternanthera pungens*, but not the morphologically related endemic species of the CSL Sierra *Alternanthera pumila*.

***Apurimacia*.** South American genus with 5 species. The endemic of the Sierras CSL *Apurimacia dolichocarpa* is the only species in Argentina. A molecular phylogeny of the tribe Millettieae (da Silva et al. 2012), where *Apurimacia* belongs, has dated the clade including the genus to ca. 1.1 Ma.

***Aristida*.** Widespread genus with ca. 300 spp. of tropical and subtropical regions of both hemispheres (Watson and Dallwitz 1992), with ca. 30 species in Argentina (Caro 1961, Sulekic 2003). The Sierras CSL endemics Aristida minutiflora var. glabriflora, *Aristida multiramea* and *Aristida sayapensis* have not been included in the phylogeny of *Aristida* by Cerros-Tlatilpa et al. (2011), that dated species included in a core South America clade to 2.37 (3.77–1.15) Ma, the most recent in the genus.

***Astragalus*.** Cosmopolitan genus with ca. 2500 species inhabiting Mediterranean arid and semiarid environments, with 61 species in Argentina (Zuloaga and Morrone 1999). Although the Sierras CSL endemic *Astragalus parodii* has not been included in molecular phylogenies, Scherson et al. (2008) showed that South American species belong to one of two clades, dated to ca. 1.89 Ma and ca. 0.98 Ma and proposed at least two migration events from North America, with a recent radiation of species in both South American clades.

***Berberis*.** Estimates of species numbers in the cosmopolitan genus *Berberis* vary between 20 (Landrum 1990) and ca. 450 (Kim et al. 2004). Thirty four species are found in Argentina (Ulloa Ulloa 2008) and the Sierras CSL endemic *Berberis hieronymi* has never been included in molecular phylogenetic studies.

***Blumenbachia*.** South American genus with ca. 12 species, 6 of them in Argentina (Zuloaga and Belgrano 2008). The Sierras CSL endemics *Berberis hieronymi* has been included in a molecular phylogeny (Hufford et al. 2005, Ackermann et al. 2006) and is a member of a clade with *Berberis insignis*, widely distributed in southern South America.

***Borreria*.** Sometimes included in *Spermacoce* L. (150 spp.), this genus comprises 18 species in Argentina. Besides the different type of fruit of *Borreria* and *Spermacoce*, the study by Groeninck et al. (2009) shows clearly that *Borreria* should be maintained as a separate genus. The Sierras CSL endemic Borreria eryngioides var. ostenii has never been included in a molecular phylogeny.

***Calydorea*.** This South American genus comprises ca. 10 species from temperate regions. There are 5 spp. in Argentina, including the Sierras CSL endemic *Calydorea undulata*. This taxon was described by Ravenna (2001) from Córdoba populations of *Calydorea pallens*; the identity of the species was verified by De Tullio et al. (2008) based on cytological and morphological evidence, but the taxon has never been included in a phylogeny.

***Carex*.** This cosmopolitan genus comprises 1500–2000 species in both Northern and Southern hemispheres (Wheeler 1990); 107 species are found in Argentina (Zuloaga et al. 2008). The Sierras CSL endemic *Carex monodynama* is found in an isolated location at the summit of the Sierra de Achala, but has not been included in molecular phylogenies.

***Cenchrus*.** Grass genus with ca. 100 species of tropical and temperate regions of both hemispheres; 14 species are found in Argentina (Gutiérrez 2012). The Sierras CSL endemic *Cenchrus rigidus* has not been included in a molecular phylogeny.

***Cerastium*.** The nearly cosmopolitan genus *Cerastium* comprises ca. 100 species with a diversity center in Eurasia and has preference for cold-temperate regions (Pedersen 1984); two migration events to North and South America have been suggested (Scheen et al. 2004). In Argentina there are 17 spp., from which the Sierras CSL endemic *Cerastium argentinum* has never been included in a molecular phylogenetic study.

***Danthonia*.** 30 species mainly from mountainous regions of the Southern Hemisphere; 7 species in Argentina (Romanutti and Anton 2012). The Sierras CSL endemic *Danthonia melanathera* has not been included in any molecular phylogeny.

***Eryngium*.** The largest genus in the Apiaceae, with about 250 species of temperate regions of all continents; 29 species are found in Argentina (Zuloaga and Morrone 1999). The Sierras CSL endemic *Eryngium agavifolium* was included in the molecular phylogeny by Calviño et al. (2008), forming a clade with *Eryngium elegans*, a widely distributed species in southern South America.

***Escallonia*.** The South American genus *Escallonia* comprises ca. 40 species of shrubs, especially in the Andes. In Argentina there are 16 species (Zuloaga et al. 2008) and the Sierras CSL endemic *Escallonia cordobensis* has been included in the phylogenetic study of Sede et al. (2013), forming a polytomy with *Escallonia petrophila*, *Escallonia ledifolia*, *Escallonia farinacea*, *Escallonia bifida* and *Escallonia laevis*, which are taxa distributed in northeastern Argentina, Brazil, Paraguay and Uruguay.

***Gentianella*.** This mostly alpine-arctic genus occurs in South America in the Andes, where is represented by ca. 150 species (von Hagen and Kadereit 2001), with about 28 species in Argentina (Zuloaga and Morrone 1999). *Gentianella* entered in South America probably more than one time, and has in the region a high rate of speciation, probably linked with the availability of suitable habitats (von Hagen and Kadereit 2001). The Sierras CSL endemic annual *Gentianella parviflora* has not been included in a molecular phylogeny.

***Geranium*.** Genus with ca. 400 species of temperate areas and tropical mountains throughout most of the world, and 18 species in Argentina (Zuloaga and Morrone 1999). There is no molecular phylogenetic study of the whole genus. Aedo et al. (2005) revised section *Andina* of the genus, to which the endemic of CSL Sierras *Geranium parodii* belongs. Aedo et al. (2005) note that *Geranium parodii* was first described as a variety of the wider distributed *Geranium sessiliflorum*, which is found in the Andes from Perú to southern Argentina and Chile.

***Geum*.** This mostly Northern Hemisphere (Smedmark and Eriksson 2002) genus comprises ca. 40 species of cold-temperate regions. In Argentina it includes 6 species, commonly found in the Andes and Patagonia. The Sierras CSL endemic *Geum brevicarpellatum* has not been included in a phylogeny.

***Gomphrena*.** This genus includes ca. 120 spp. of tropical regions, with 38 species in Argentina (Borsch 2008b). Gomphrena pulchella subsp. rosea and Gomphrena pulchella var. bonariensis are restricted to the Sierras CSL, but *Gomphrena pulchella* is widely distributed in southern South America. There is no molecular phylogenies including these taxa.

***Grindelia*.** A New World temperate genus with 73 species in western North America and southern South America (Sancho and Ariza Espinar 2003); 19 species in Argentina (Freire 2008). There are two endemic taxa in the Sierras CSL, Grindelia cabrerae var. alatocarpa and *Grindelia globularifolia*. The latter was included in the phylogeny by Moore et al. (2012), and resolved within the South American clade.

***Gymnocalycium*.** South American genus of ca. 50 species, mostly in mountain ranges of Argentina; with 16 endemic species and subspecies in the Sierras CSL, is the genus with largest number of endemic species in the region (Demaio 2012). The phylogeny of the genus by Demaio et al. (2011) recovered three clades (subgenera) living sympatrically in the Sierras CSL: *Scabrosemineum* (6 endemic taxa); *Gymnocalycium* (9 endemic taxa); and *Trichomosemineum* (1 taxon). Divergence times in Cactaceae (Arakaki et al. 2011) showed that the differentiation of the genus might have occurred between the Miocene and Pliocene (7.5–6.5 Ma); Hernández-Hernández et al. (2014) gave a younger date of 5.08 (3.09–7.55) Ma.

***Habranthus*.** American genus of ca. 30 species, mostly South American but with five species in North America, probably introduced (Roitman et al. 2007). Twenty three species grow in Argentina (Zuloaga et al. 2008). The Sierras CSL endemic *Habranthus sanavironae* is similar in flowers size to *Habranthus robustus* (=*Zephyranthes robusta*) (Roitman et al. 2007), which is widespread in Central Argentina and Southern Brazil. *Habranthus sanavironae* has never been included in a phylogeny.

***Helenium*.** American genus of ca. 40 species, mostly southern USA and Mexico (Bremer 1994); in Argentina three species (Novara and Petenatti 2000). The Sierras CSL endemic *Helenium argentinum* has never been included in a molecular phylogeny.

***Hieracium*.** Nearly cosmopolitan genus with ca. 1000 species (Bremer 1994); 45 species in Argentina (Cerana and Ariza Espinar 2003). Presence of polyploidy, mixed breeding systems and apomixis (Chrtek et al. 2009) complicate its systematics and make estimation of taxon numbers highly variable. None of the Sierras CSL endemics (*Hieracium achalense*, *Hieracium cordobense* and *Hieracium criniceps*) have been included in molecular phylogenetic studies.

***Hypochaeris*.** This genus with ca. 60 species occurs in Europe, Asia and North Africa, and South America, while the greatest number of species is found in the latter (Bremer 1994); 30 species are found in Argentina (Bortiri 1999). Studies by Samuel et al. (2003) and Tremetsberger et al. (2005) did not include the Sierras CSL endemic *Hypochaeris caespitosa*.

***Hysterionica*.** South American genus with 12 species in Brazil, Uruguay and Argentina; 9 species are found in Argentina (Freire 2008). The Sierras CSL endemics Hysterionica dianthifolia var. dianthifolia and Hysterionica dianthifolia var. pulvinata have not been included in molecular phylogenies; Noyes and Rieseberg (1999) related this genus with *Conyza* and *Erigeron*, both of the Northern hemisphere.

***Isostigma*.** Small South American genus with 11 species from subtropical areas; 5 species are found in Argentina (Peter 2009). The Sierras CSL endemic *Isostigma cordobense* has not been included in a molecular phylogeny.

***Junellia*.** South American genus with 36 species distributed in Perú, Bolivia, Chile and the most in Argentina. The molecular phylogeny of O´Leary et al. (2009) did not include *Junellia bisulcata*, whose variety *campestris* is endemic of the Sierras CSL. *Junellia bisulcata* has a wide geographical distribution in Andean and sub-Andean ranges of northern Argentina, northern Chile and southern Bolivia.

***Melica*.** Grass genus with 80 species from temperate regions of both hemispheres; 17 species in Argentina (Morrone and Zuloaga 2012). The Sierras CSL endemic *Melica decipiens* has not been included in any phylogenetic study.

***Mimosa*.** This genus comprises ca. 480 spp. of tropical and warm zones of the American continent; in Argentina there are 55 species (Zuloaga and Morrone 2012). The genus is rich in narrow endemics (Simon et al. 2011). *Mimosa cordobensis* has not been included in a phylogenetic study.

***Mostacillastrum*.** This South American genus comprises 17 species distributed from southern Peru and Bolivia to northern Patagonia (Al-Shehbaz 2006); *Mostacillastrum carolinense* was described originally as a *Sisymbrium* (Scappini et al. 2004). The phylogeny by Warwick et al. (2009) included other *Mostacillastrum* species but not *Mostacillastrum carolinense*; the tribe Thelypodieae where *Mostacillastrum* belongs shows low molecular differentiation.

***Mutisia*.** Excepting for a few species growing in southern Brazil and adjacent regions of Paraguay and Uruguay, most of the 59 species of this genus are found in the Andes (Cabrera 1971). Argentina has 35 species (Freire 2008) and the Sierras CSL endemic Mutisia castellanosii var. comechingoniana has never been included in a phylogenetic study.

***Nassella*.** Grass genus with ca. 80 species distributed in the American continent, especially in the Andes (Mabberley 1997). Due to different generic concepts, the species number in Argentina varies between 16 (Rosa et al. 2005) and 70 (Cialdella 2012). The Sierras CSL endemic *Nassella stuckertii*, related to the widespread *Nassella tenuissima*, has not been included in molecular phylogenies.

***Nothoscordum*.** This mostly South American genus comprises more than 70 species, with 39 in Argentina and a single species, *Nothoscordum gracile*, distributed through the Americas (Zuloaga et al. 2008; Rodrigues Souza et al. 2012). The Sierras CSL endemic *Nothoscordum achalense* has not been included in a phylogenetic study.

***Parodianthus*.** Small genus with only two known species, restricted to central Argentina. *Pardianthus capillaris* grows only in the northern extreme of the Sierras CSL. Marx et al. (2010) showed *Parodianthus* formed a clade with *Casselia* and *Tamonea* in agreement with previous morphological studies (Martínez and de Romero 2003), but did not include the Sierras CSL endemic *Parodianthus capillaris*. *Casselia* is distributed in Brazil, Bolivia, and Paraguay, while *Tamonea* is widespread from Mexico and the Caribbean to Brazil and Paraguay.

***Plantago*.** The ca. 260 species of *Plantago* are distributed worldwide (Dunbar-Co et al. 2008); in Argentina there are 34 species (Zuloaga et al. 2008). The Sierras CSL endemic *Plantago densa* has never been included in a molecular phylogeny.

***Poa*.** The largest genus of the Poaceae, with a number varying between 500–575 species distributed in all temperate-cold regions of the world (Gillespie and Soreng 2005; Gillespie et al. 2007). There are 62 species in Argentina (Giussani et al. 2012), and from the two Sierras CSL endemics, *Poa hubbardiana* and *Poa stuckertii*, only the latter has been included in a phylogeny (Gillespie et al. 2007), where it was placed together with the North American *Poa arachnifera*.

***Portulaca*.** Distributed worldwide, this genus comprises ca. 100 species, mainly in the tropics and subtropics, with centers of diversity in South America and Africa. There are 29 taxa in Argentina, including the Sierras CSL endemic Portulaca confertifolia var. cordobensis (Zuloaga et al. 2008). A recent molecular phylogeny included *Portulaca confertifolia* (Ocampo and Columbus 2011) and showed the node including this species is dated to 3 Ma.

***Prosopis*.** This genus comprises 45 species of warmer regions of America, Southeast Asia and Africa. There are 28 spp. in Argentina (Zuloaga and Morrone 1999), and the Sierras CSL endemic *Prosopis campestris* has been included in the phylogenetic study by Catalano et al. (2008). The study shows a probable divergence time during the late Pliocene (1.8 Ma).

***Senecio*.** One of the most species-rich genera of the Asteraceae, *Senecio* has ca. 3000 species distributed all over the world. In Argentina there are 423 species (Freire 2008), with regions of highest diversity the Andes and Patagonia (Cabrera 1971). The two Sierras CSL endemics, *Senecio achalensis* and *Senecio retanensis* have never been included in any phylogenetic study.

***Siphocampylus*.** South American genus with ca. 220 species, 16 growing in mountainous regions of Argentina. Neither endemic variety of *Siphocampylus foliosus* endemic to the Sierras CSL has been included in any phylogenetic study.

***Solanum*.** Sub-cosmopolitan genus with around 1400 species of warm regions of the world. In Argentina there are 115 species and three hybrids (Barboza 2013). The three Sierras CSL endemics, *Solanum concarense*, *Solanum ratum* and *Solanum restrictum* have not been included in phylogenetic studies; *Solanum concarense* has been accepted by Barboza (2013), but *Solanum restrictum* and *Solanum ratum* were treated as synonyms of *Solanum salicifolium*, an extremely variable species distributed in western Argentina and Bolivia.

***Soliva*.** Small and mostly South American genus, it also has widespread species that occur in both Australia and North America. 5 species grow in Argentina (Zuloaga et al. 2008). The molecular phylogeny of the tribe Anthemideae by Watson et al. (2000) did not include the Sierras CSL endemic *Soliva triniifolia* but *Solanum anthemifolia*, a widespread species occurring in adjacent areas.

***Sophora*.** Cosmopolitan genus with ca. 45 species; 2 species in Argentina. *Sophora linearifolia* is endemic to the Sierras CSL, but has not been included in the phylogeny by Mitchell and Heenan (2002), although it was mentioned as closely related to coastal Chilean species belonging to Sect. Edwardasia that also includes species from the Pacific islands and New Zealand (Crowder 1982; Peña et al. 2000).

***Sphaeralcea*.** This genus has ca. 40 herbaceous and shrubby species occurring in temperate parts of the Americas (Krapovickas 1965, 1970). The Sierras CSL endemic small shrub *Sphaeralcea cordobensis* has been included in the phylogeny of *Tarassa* by Tate and Simpson (2003). *Sphaeralcea cordobensis* is a diploid included in a polyphyletic assemblage with *Tarassa* and *Nototriche*; however the unique morphology and geographic distribution suggest the three genera are different lineages (Tate and Simpson 2003).

***Tillandsia*.** Pan-American genus with ca. 550 species (Barfuss et al. 2005). *Tillandsia xiphioides* is widely distributed in southern South America, and was included in the analysis of Barfuss et al. (2005); it joined an Andean clade forming a polytomy and characterized by its rapid evolution (Barfuss et al. 2005: 347).

***Trichloris*.** Grass genus with 2 disjunct species distributed in north-central Argentina and Bolivia and Mexico and southern USA (Rúgolo and Molina 2012). The endemic Trichloris pluriflora f. macra has not been included in a molecular phylogeny.

***Trichocline*.** Genus of 22 species, most of them in South America from southern Peru to central Argentina and Chile (Katinas et al. 2008), with 13 species in Argentina (Zuloaga et al. 2008). *Trichocline plicata*, endemic to the Sierras CSL, has not been included in molecular phylogenies; a widespread and related species, *Trichocline reptans*, grows in sympatry.

***Tridens*.** Grass genus with 14 species distributed in tropical and temperate regions of the Americas; 3 species in Argentina (Romanutti and Anton 2012). The Sierras CSL endemic *Tridens nicorae* has not been studied in molecular phylogenetic studies.

***Valeriana*.** This genus comprises ca. 350 species usually found in mountainous regions (Bell and Donoghue 2005), while 81 are found in Argentina (Kutschker 2008). The roughly 175 South American species form a clade suggesting the existence of a modern center of diversification in the Andes (Bell and Donoghue 2005, Bell et al. 2012). Neither of these works has included the Sierras CSL endemics *Valeriana ferax* and *Valeriana stuckertii*.

***Zephyranthes*.** This genus comprises about 65 Neotropical species. The molecular phylogeny of American Amaryllidaceae by Meerow et al. (2000) showed the genus as polyphyletic, with two well differentiated clades including South American taxa. *Zephyranthes longystila*, the endemic species of Sierras CSL, was not included in this work.

### Endemic taxa of Sierras CSL and widespread related taxa

A total of 28 taxa of the endemics of the Sierras CSL is sympatric with a widespread congener, or with one found close to the area (Table 4).

**Table 4.** Sympartry/parapatry of endemic taxa of Sierra CSL and widespread congeners.

Endemic taxa Sierra CSL	Widespread related taxa	Source
Adesmia cordobensis var. appendiculata	*Adesmia cordobensis* Burkart	Zuloaga and Morrone 1999
*Alternanthera pumila*	*Alternanthera pungens* Kunth	Zuloaga and Morrone 1999
Aristida minutiflora var. glabriflora	*Aristida minutiflora* Caro	Zuloaga and Morrone 1999
*Blumenbachia hieronymi*	*Blumenbachia insignis* Schrad.	Hufford et al. 2005, Ackerman et al. 2006
Borreria eryngioides var. ostenii	*Borreria eryngioides* Cham. & Schltdl.	Zuloaga and Morrone 1999
*Calydorea undulata*	*Calydorea pallens* Briseb.	Zuloaga and Morrone 1999
*Eryngium agavifolium*	*Eryngium elegans* Cham. & Schltdl.	Calviño et al. 2008
*Escallonia cordobensis*	*Escallonia petrophila* Rambo & Sleumer, *Escallonia ledifolia* Sleumer, *Escallonia farinacea* A. St.-Hil., *Escallonia bifida* Link & Otto, *Escallonia laevis* (Vell.) Sleumer, *Escallonia hypoglauca* Herzog and *Escallonia tucumanensis* Hosseus	Sede et al. 2013
*Geranium parodii*	*Geranium sessiliflorum* Cav.	Aedo et al. 2005
Gomphrena pulchella subsp. rosea	*Gomphrena pulchella* Mart.	Borsch 2008
Grindelia cabrerae var. alatocarpa	*Grindelia cabrerae* Ariza	Zuloaga and Morrone 1999
*Grindelia globularifolia*	*Gomphrena pulchella* Mart.	Moore et al. 2012
*Habranthus sanavironae*	*Habranthus robustus* Herb. ex Sweet	Roitman et al. 2007
Junellia bisulcata var. campestris	*Junellia bisulcata* (Hayek) Moldenke	O’Leary 2009
Mutisia castellanosii var. comechingoana	*Mutisia castellanosii* Cabrera	Zuloaga and Morrone 1999
*Nassella stuckertii*	*Nassella tenuissima* (Trin.) Barkworth	Cialdella 2012
*Parodianthus capillaris*	*Parodianthus illicifolium* (Moldenke) Tronc.	Marx et al. 2010
*Portulaca confertifolia*	*Portulaca eruca* Hauman, *Portulaca perennis* R.E. Fr., *Portulaca mucronulata* D. Legrand, *Portulaca obtusa* Poelln. and *Portulaca gilliesii* Hook.	Ocampo and Columbus 2011
*Prosopis campestris*	*Prosopis chilensis* (Molina) Stuntz emend. Burkart	Catalano et al. 2008
Siphocampylus foliosous var. glabratus; Siphocampylus foliosus var. minor	*Siphocampylus foliosus* Griseb.	Zuloaga and Morrone 1999
*Solanum concarense*, *Solanum ratum*, *Solanum restrictum*	*Solanum salicifolium* Phil.	Knapp 2013
*Soliva triniifolia*	*Soliva anthemifolia* (Juss.) Sweet	Zuloaga and Belgrano 2008
*Sphaeralcea cordobensis*	*Sphaeralcea crispa* Baker f.	Tate and Simpson 2003
Tillandsia xiphioides var. minor	*Tillandsia xiphioides* Ker Gawl.	Zuloaga and Morrone 1999
Trichloris pluriflora fo. macra	*Trichloris pluriflora* E. Fourn.	Rúgolo and Molina 2012
*Trichocline plicata*	*Trichocline reptans* (Wedd.) Hieron.	Zuloaga and Belgrano 2008

## Discussion

### Recent origins of endemism in the Sierras CSL

Two main sources of evidence suggest that 46 taxa (ca 40.4%) of the endemics of the Sierras CSL are neoendemic taxa *sensu* Stebbins and Major (1965). The first evidence arises from available molecular phylogenetic studies (Table 3), which show 10 taxa (11.24 %) included in clades with divergence times of ca. 5 Ma or less. The second source is the existence of sympatry between an endemic taxon of the Sierras and a widespread taxon of the same genus (Table 4). *Acanthocalycium spiniflorum* was included in the study by Hernandez-Hernandez et al. (2014), showing a divergence time of ca. 2.5 Ma. Ackerman et al. (2006) included *Blumenbachia hieronymii* in their phylogeny and it was resolved in a clade with *Blumenbachia insignis*, which is widely distributed in southern South America. *Eryngium agavifolium*, included in the phylogeny by Calviño et al. (2008) joined in a well-supported clade with *Eryngium elegans*, which is widely distributed in southern South America. *Escallonia cordobensis* was included in the phylogeny by Sede et al. (2013), forming an unresolved clade with *Escallonia petrophila*, *Escallonia ledifolia*, *Escallonia farinacea*, *Escallonia bifida* and *Escallonia laevis*, *Escallonia hypoglauca* and *Escallonia tucumanensis*. All these species are barely differentiated (Sede et al. 2013: 173), which suggests that the group evolved realtively recently. *Grindelia globularifolia* shows a similar pattern in the phylogeny by Moore et al. (2012), grouped in a large polytomy with several widespread species. The phylogram of *Gymnocalycium* by Demaio et al. (2011) showed that *Gymnocalycium saglionis* is the first branching taxon in the genus. Hernández-Hernández et al. (2014) showed that *Gymnocalycium saglionis* diverged ca. 5 Ma, and the clade including a species of the subgenus *Scabrosemineum* (*Gymnocalycium guanchinense*) - where many species of the Sierras CSL belong - diverged ca. 2.5 Ma. In *Portulaca*, the phylogeny by Ocampo and Columbus (2011) set a divergence time for *Portulaca confertifolia* of ca. 3 Ma. *Prosopis campestris* was included in the chronogram of Catalano et al. (2008), with a divergence time of ca. 1.8 Ma. *Sphaeralcea cordobensis* was included in the phylogeny by Tate and Simpson (2003), forming a clade with the widely distributed *Sphaeralcea crispa*. *Tillandsia xiphioides* has been included in the molecular phylogeny of Barfuss et al. (2005), who suggested all taxa of *Tillandsia* to be phylogenetically young, as inferred by the low genetic divergence. Tillandsia xiphioides var. minor was a member of a polytomy in their phylogenetic reconstruction, suggesting that it had not time to undergo a complete differentiation.

The second source of supporting evidence is the existence of pairs of taxa with the endemic species of Sierras CSL occurring in sympatry or parapatry with a widespread congeneric species. Walck et al. (2001) compared *Solidago shortii* Torr. & A.Gray, a narrow endemic species of eastern North America, with *Solidago altissima* L., a widespread species, and found that *Solidago altissima* is a better competitor than *Solidago shortii* because of its greater height, larger leaf area and more extensive clonal growth. On the other hand, *Solidago shortii* tolerates drought stress better than *Solidago altissima* because the allocation of a higher percentage of biomass to roots, higher root/shoot ratio and greater capacity to maintain leaf turgor under xeric conditions. As a consequence of the differences in these traits, and although the lack of a molecular phylogenetic framework precludes conclusive classification, Walck et al. (2001) suggested the endemic taxon to be probably derived from the widespread one.

These aspects of the endemics of the Sierras (inclusion in clades and sympatry with a widespread congeneric taxon) are congruent with the geological and biological history of the region. The Sierras CSL system is the result of a ca. 520 Ma (Paleozoic) orogenic process that around 399 Ma was subject to an intrusion of magmatic batholiths (Baldo et al. 1996). The current arrangement, with blocks of basement tilted eastwards, is the result of the Andean orogeny, which rejuvenated the whole region in the Miocene-Pliocene, starting at ca. 5.3 Ma (Baldo et al. 1996). The actual composition of the vegetation of the Sierras CSL would have been assembled during this later interval, and has probably been preceded by times of major interchange with neighboring areas (Prado 1993a).

### Altitudinal distribution of endemic taxa

The distribution of endemic taxa varied among the altitudinal belts. In a chorological study on 20 selected sites of the Sierras CSL, Cabido et al. (1998) emphasized that the upper vegetation belt in the Sierras CSL is distinct not only because its richness in Andean phytogeographic elements, but also due to the occurrence of highly restricted endemics. The data presented here show that the altitudinal belt with highest number of endemic taxa is the lowest (the sierra forest belt) with 35 endemic taxa, while the upper (the high-altitude grasslands and woodlands) has 11 endemic taxa (Fig. 2, Table 2). The cumulative number of endemic taxa in the two lower belts suggests that differentiation and establishment of neoendemic taxa occurred most probably in the lower vegetation belts of the Sierras CSL, which have clear floristic affinities with surrounding Chaco vegetation (Prado et al. 1993a, 1993b; Cabido et al. 1998).

## Conclusion

### Why more studies on local endemics are needed

Our data suggests that many endemic taxa of the Sierras de Córdoba and San Luis have developed as consequence of differentiation processes occurred during the last approximately 7 Ma. Likewise, the whole flora of the Sierras has been only partially isolated from surrounding Chaco vegetation. The overall lower presence of endemic taxa of the Sierras in phylogenetic studies emphasizes the need for their inclusion in such studies as a mean to achieve a better understanding of the evolutionary and biogeographical history of this area. Lastly, the present work also suggests that, although extracting information on speciation from phylogenies is not straightforward (Barraclough and Nee 2001), including endemic taxa in phylogenetic studies could provide useful insights on evolution of endemism and areas of endemism. Although our analysis is specifically aimed at a defined geographic area, the concept of analyzing all the endemic taxa of a particular zone could reveal patterns of biodiversity, since endemic taxa richness is a product of the interaction between historical processes as speciation or migration and contemporary factors as ecology or landscape use.
